# Clinical and health care utilization variables can predict 90-day hospital re-admission in adults with Crohn’s disease for point of care risk evaluation

**DOI:** 10.1186/s12876-024-03226-7

**Published:** 2024-05-17

**Authors:** C Dziegielewski, S Gupta, J Begum, M Pugliese, J Lombardi, Kelly E, McCurdy JD, R Sy, Saloojee N, Ramsay T, Benchimol EI, Murthy SK

**Affiliations:** 1 Department of Medicine, University of Ottawa, Ottawa, ON Canada; 2 Department of Medicine, University of Toronto, Toronto, Ontario, ON Canada; 3 Ottawa Hospital Research Institute, University of Ottawa, Ottawa, ON Canada; 4 ICES uOttawa, Ottawa, ON Canada; 5 Department of Medicine, McMaster University, Hamilton, Ontario, ON Canada; 6 The Ottawa Hospital IBD Centre, 501 Smyth Rd, K1H 8L6 Ottawa, ON Canada; 7 SickKids Inflammatory Bowel Disease Centre, Division of Gastroenterology, Hepatology and Nutrition, The Hospital for Sick Children, Toronto, ON Canada; 8 Child Health Evaluative Sciences, SickKids Research Institute, Toronto, ON Canada; 9 ICES, Toronto, ON Canada; 10 Department of Paediatrics and Institute of Health Policy, Management and Evaluation, University of Toronto, Toronto, ON Canada

**Keywords:** Crohn’s disease, Re-hospitalization, Multivariable models

## Abstract

**Background:**

Hospital re-admission for persons with Crohn’s disease (CD) is a significant contributor to morbidity and healthcare costs. We derived prediction models of risk of 90-day re-hospitalization among persons with CD that could be applied at hospital discharge to target outpatient interventions mitigating this risk.

**Methods:**

We performed a retrospective study in persons with CD admitted between 2009 and 2016 for an acute CD-related indication. Demographic, clinical, and health services predictor variables were ascertained through chart review and linkage to administrative health databases. We derived and internally validated a multivariable logistic regression model of 90-day CD-related re-hospitalization. We selected the optimal probability cut-point to maximize Youden’s index.

**Results:**

There were 524 CD hospitalizations and 57 (10.9%) CD re-hospitalizations within 90 days of discharge. Our final model included hospitalization within the prior year (adjusted odds ratio [aOR] 3.27, 95% confidence interval [CI] 1.76–6.08), gastroenterologist consultation within the prior year (aOR 0.185, 95% CI 0.0950–0.360), intra-abdominal surgery during index hospitalization (aOR 0.216, 95% CI 0.0500–0.934), and new diagnosis of CD during index hospitalization (aOR 0.327, 95% CI 0.0950–1.13). The model demonstrated good discrimination (optimism-corrected c-statistic value 0.726) and excellent calibration (Hosmer-Lemeshow goodness-of-fit p-value 0.990). The optimal model probability cut point allowed for a sensitivity of 71.9% and specificity of 70.9% for identifying 90-day re-hospitalization, at a false positivity rate of 29.1% and false negativity rate of 28.1%.

**Conclusions:**

Demographic, clinical, and health services variables can help discriminate persons with CD at risk of early re-hospitalization, which could permit targeted post-discharge intervention.

**Supplementary Information:**

The online version contains supplementary material available at 10.1186/s12876-024-03226-7.

## Background

Inflammatory bowel disease (IBD), which encompasses Crohn’s disease (CD) and ulcerative colitis (UC), is characterized by chronic and/or recurrent bouts of inflammation of the gastrointestinal tract and extra-intestinal organs. IBD prevalence is rising worldwide, with prevalence of over 0.3% in North America and much of Europe [1]. Many individuals with IBD incur recurrent hospitalizations relating to disease flares and/or complications, often necessitating surgery or prolonged hospital stays, which are associated with substantial morbidity and health care costs [2–4]. The cumulative rate of hospitalization in persons with CD in European populations is nearly 40% [5]. In Canada, close to 20% of adults with CD are hospitalized annually [2]. Up to 40% of CD-related hospitalizations are associated with re-admissions within a year, with risk factors including younger age, chronic pain, penetrating or perianal disease, steroid or immunomodulator exposure, and need for surgery [6–10]. Individuals requiring re-admission often have greater co-morbidity burden and require longer length of stay, resulting in greater associated healthcare costs [11]. Reducing the risk of recurrent hospitalizations through timely outpatient intervention could help lower patient morbidity and healthcare costs.

There are currently no risk assessment models to help predict which CD patients are at increased risk of early re-admission. Re-admission rates at 30 and 90 days have been shown to be a metric for quality of care for persons with chronic diseases and potentially preventable hospitalizations [12]. This highlights the importance of recognizing individuals who are at high risk of early re-hospitalization, in order to target intensive post-discharge outpatient interventions that may prevent this outcome. Previous studies have identified several predictors of CD-related hospitalizations, including younger age, greater disease extent and severity, and steroid and/or immunomodulator exposure, as well as predictors of re-admission, including younger age, penetrating disease, chronic pain, opioid and steroid use [7–10, 13–17]. A multivariable model combining multiple factors that impact risk of hospital re-admission could be incorporated into clinical practice as a bedside tool to better predict which patients are at highest risk of early re-admission. We aimed to develop a multivariable risk prediction model of 90-day hospital re-admission among persons with CD, as early outpatient intervention may be feasible in such individuals to reduce the risk of post-discharge disease relapse.

## Methods

### Study cohort and data sources

We conducted a retrospective cohort study of all adults (age ≥ 18 years) with a new or established diagnosis of CD admitted to The Ottawa Hospital for an IBD flare or IBD-related intestinal or perianal complication (excluding bowel cancer) between April 1, 2009 and March 31, 2016. The Ottawa Hospital is a tertiary care hospital and regional IBD referral center, serving a population of more than 1.2 million people. We identified potential participants through the Ottawa Hospital Data Warehouse, a repository of hospitalizations, emergency department visits, day surgery visits (including endoscopy) and investigations (including laboratory data, pathology and diagnostic imaging) occurring at The Ottawa Hospital. We queried all adult persons with one or more hospital encounters associated with a discharge diagnosis of CD (International Classification of Diseases (ICD), 10th Version (ICD-10) diagnostic code K50.x), UC (ICD-10 code K51.x), “noninfective gastroenteritis and colitis, unspecified” (ICD-10 code K52.9) or “indeterminate colitis” (ICD-10 code K52.3). We manually reviewed the medical records of these patients to identify eligible hospitalizations for a CD-related indication.

Following confirmation of diagnosis and data collection on candidate predictors through chart review, we deterministically linked study patients to province-wide health administrative datasets for Ontario, Canada, to ascertain regional hospital re-admissions for an IBD-related indication across Ontario within 90 days of index hospitalization, as well as to ascertain additional candidate predictor variables. These data were linked using unique encoded identifiers and analyzed at ICES, an independent, non-profit research institute whose legal status under Ontario’s health information privacy law allows it to collect and analyze health care and demographic data, without consent, for health system evaluation and improvement. Administrative, clinical and demographic information pertaining to hospitalizations across Canada are comprehensively captured in the Canadian Institutes of Health Information Discharge Abstract Database (CIHI-DAD) [18]. We defined hospital admission for an IBD-related indication in the CIHI-DAD as one that reported a most-responsible, co-morbid, or hospital transfer diagnosis, which significantly impacted health resource utilization, on the discharge abstract that was compatible with an IBD-specific diagnosis (ICD-10 K50.x, K51.x), as per previous studies [19, 20]. We included the diagnosis codes for ulcerative colitis to avoid excluding hospitalizations with diagnostic misclassification in health administrative data.

Following linkage, we excluded individuals who did not have valid and continuous health care registration in Ontario for at least 1 year prior to the date of admission or 90 days following the date of discharge, as these time periods were necessary to ascertain several predictor variables and the outcome, respectively. We also excluded any individuals without a valid ICES identification number, which is necessary for person linkage across datasets.

The study protocol was approved by the Research Ethics Boards of the Ottawa Health Sciences Network (Ottawa, Canada) and ICES Privacy. The use of the data collected by ICES in this project is authorized under section 45 of Ontario’s Personal Health Information Protection Act (PHIPA) and does not require review by a Research Ethics Board.

### Candidate predictors and outcomes

Candidate variables were selected a priori based on literature review and author consensus. We ascertained candidate predictors for eligible participants through chart review and linkage to health administrative datasets. We also used established macros at ICES to define the Charlson co-morbidity score and rural-urban status of residence for each individual at the time of index hospital admission [21]. The complete candidate predictor list, encompassing demographic, disease, treatment and health services factors that could impact CD prognosis, is provided in Supplemental Table 1.

We defined our outcome as 90-day hospital re-admission for a CD-related indication as this outcome has a high probability of being related to the disease flare or complication that led to the initial hospitalization and is potentially preventable through expedited outpatient monitoring and intervention following hospital discharge. As all Canadians are publicly insured to access health services anywhere in the country, hospital re-admissions occurring outside Ontario would not have been captured in ICES datasets; we anticipate that less than 5% of IBD-related re-hospitalizations in this study would have fallen into that category.

### Analytic methods

We performed stepwise multivariable logistic regression to model 90-day CD-related hospital re-admission. Each candidate predictor that had a significant bivariate association with the outcome at a p-value of < 0.2 would enter the model (in order of ascending p-value) and a variable would be retained in the model if it maintained an independent association with the outcome at a p-value of < 0.1 in the final model. All variables were tested for multicollinearity and only one out of a set of collinear variables was tested for inclusion in the model.

We assessed overall model performance based on the discriminatory capacity (c-statistic) and calibration (Hosmer-Lemeshow goodness-of-fit test). We further assessed sensitivity, specificity, positive predictive value (PPV), negative predictive value (NPV), positive likelihood ratio (PLR), negative likelihood ratio (NLR), false positive rate (FPR), and false negative rate (FNR) for model prediction of 90-day hospital-readmission based on a predicted probability cut point that was pre-specified to optimize Youden’s (J) index [22]. The FPR is the proportion of individuals who did not undergo re-admission who were incorrectly predicted to be at high risk of readmission by the model; conversely, the FNR is the proportion of individuals who were ultimately re-admitted within 90 days who were incorrectly predicted to be at low risk of readmission by the model. We performed bootstrap internal validation, using 200 bootstrapped samples, to derive the “optimism-corrected” c-statistic value (which downgrades model performance to approximate expected discriminatory capacity on external validation). We used SAS 9.4. as our statistical software to perform this analysis.

## Results

Out of an initial 1,649 hospitalizations that were identified from our initial query of the Data Warehouse, 524 hospitalizations were confirmed to have occurred in persons with CD for a disease flare or CD-related complication and met all study eligibility criteria. From this cohort, 57 (10.9%) persons were associated with hospital re-admission in Ontario for a CD-related indication within 90 days of discharge. Baseline and disease characteristics of the study cohort are summarized in Table 1. The mean age of CD diagnosis was 30.6 ± 16.0 years and 44.3% of the cohort was male. More than 40% of the cohort had a history of fibrostenotic or penetrating complications, more than one-third had undergone prior intestinal surgery and more than 40% had received prior biologic therapy. During index hospitalization, 10% of the cohort were newly diagnosed with CD, 8.1% suffered an intra-abdominal catastrophe (intestinal perforation, toxic megacolon, fulminant colitis, or intra-abdominal sepsis), 13.7% underwent intra-abdominal surgery, and 2.5% were admitted to the ICU (intensive care unit). Biologic prescription at discharge was observed in 33.4% of hospitalizations.

**Table 1:** Baseline Characteristics Among Persons with CD Relative to Timing of Index Hospitalization

Variable	Total cohort (*n* = 524)	Not Re-hospitalized (*n* = 467)	Re-hospitalized (*n* = 57)
**Pre-Admission Characteristics**			
Sex, n (%)			
Male Female	232 (44.3%) 292 (55.7%)	203 (43.5%) 264 (56.4%)	29 (50.9%) 28 (49.1%)
Age at CD Diagnosis (years; mean ± SD)	30.6 ± 16.0	30.6 ± 16.3	30.8 ± 14.1
Disease Duration (years; mean ± SD)	9.96 ± 10.8	9.80 ± 10.7	11.2 ± 11.3
Disease Distribution, n (%)			
Missing Ileal Ileocolonic Colonic	21 (4.0%) 197 (37.6%) 171 (32.6%) 135 (25.8%)	15 (3.2%) 177 (37.9%) 148 (31.7%) 127 (27.2%)	6 (10.5%) 20 (35.1%) 23 (40.4%) 8 (14.0%)
History of Fibrostenotic/Penetrating Disease, n (%)			
Missing No Yes	5 (1.0%) 303 (57.8%) 216 (41.2%)	4 (0.9%) 274 (58.7%) 189 (40.5%)	1 (1.8%) 29 (50.9%) 27 (47.4%)
History of Perianal Fistulizing Disease or Perianal Surgery, n (%)			
No Yes	398 (76.0%) 126 (24.0%)	353 (75.6%) 114 (24.4%)	45 (78.9%) 12 (21.1%)
History of Bowel Resection, n (%)			
Missing No Yes	4 (0.8%) 334 (63.7%) 186 (35.5%)	4 (0.9%) 304 (65.1%) 159 (34.0%)	0 (0.0%) 30 (52.6%) 27 (47.4%)
History of Extra-intestinal manifestations, n (%)			
Missing No Yes	6 (1.1%) 411 (78.4%) 107 (20.4%)	5 (1.1%) 366 (78.4%) 96 (20.6%)	1 (1.8%) 45 (78.9%) 11 (19.3%)
Past Exposure to Steroids, n (%)			
Missing No Yes	6 (1.1%) 251 (47.9%) 267 (51.0%)	6 (1.3%) 231 (49.5%) 230 (49.3%)	0 (0.0%) 20 (35.1%) 37 (64.9%)
Past Exposure to Immunomodulator, n (%)			
Missing No Yes	5 (1.0%) 230 (43.9%) 289 (55.2%)	5 (1.1%) 203 (43.5%) 259 (55.5%)	0 (0.0%) 27 (47.4%) 30 (52.6%)
Past Exposure to Biologic, n (%)			
Missing No Yes	5 (1.0%) 306 (58.4%) 213 (40.6%)	5 (1.1%) 274 (58.7%) 188 (40.3%)	0 (0.0%) 32 (56.1%) 25 (43.9%)
CD Hospitalization Within Prior Year, n (%)			
No Yes	404 (77.1%) 120 (22.9%)	372 (79.7%) 95 (20.3%)	32 (56.1%) 25 (43.9%)
Gastroenterologist Visit Within Prior Year, n (%)			
No Yes	242 (46.2%) 282 (53.8%)	198 (42.4%) 269 (57.6%)	44 (77.2%) 13 (22.8%)
***Hospital Admission Characteristics***			
Age at Index Hospitalization (years; mean ± SD)	40.7 ± 16.5	40.5 ± 16.6	42.1 ± 15.8
New CD Diagnosis During Index Hospitalization, n (%)			
No Yes	461 (88.0%) 63 (12.0%)	--	--
Admitting Service, n (%)			
Gastroenterology Other Medical Specialty Surgery	189 (36.1%) 133 (25.4%) 202 (38.5%)	173 (37.0%) 117 (25.0%) 177 (37.9%) %	16 (28.1%) 16 (28.1%) 25 (43.9%)
Length of Hospital Stay (days; mean ± SD)	8.94 ± 11.6	9.13 ± 11.8	7.39 ± 8.86
n (%)			
No Yes	511 (97.5%) 13 (2.5%)	--	--
n (%)			
	429 (91.9%) 38 (8.1%)	--	--
n (%)			
	452 (86.3%) 72 (13.7%)	--	--
***Hospital Discharge Characteristics***			
Discharge with Biologic, n (%)			
Missing No Yes	4 (0.9%) 307 (65.7%) 156 (33.4%)	0 (0.0%) 43 (75.4%) 14 (24.6%)	4 (0.8%) 350 (66.8%) 170 (32.4%)

Notes: Intra-abdominal catastrophe is defined as either abdominal perforation, toxic megacolon, fulminant colitis, or intra-abdominal sepsis. ICU = intensive care unit --Not reported due to small number of events in one or more groups

Re-hospitalization for an acute CD-related indication within 90 days of discharge was observed more often among individuals with the following characteristics prior to index hospitalization: male sex, established diagnosis of CD, ileal involvement, history of fibrostenotic or penetrating complications, prior bowel resection, prior exposure to immunosuppressive therapy CD-hospitalization within the prior year, and absence of gastroenterologist consultation within the prior year. Re-admission within 90 days was also observed more often among individuals with the following characteristics during index hospitalization: admission to a gastroenterology service, admission for a simple intestinal disease flare, intra-abdominal surgery, discharge narcotic prescription, and absence of discharge biologic prescription.

A comprehensive list of the candidate predictors that were tested in the multivariable models of 90-day hospital re-admission, along with their bivariate associations with the outcome, is presented in Supplemental Table 1. In total, 42 candidate predictors were tested, of which 12 ultimately met criteria for further testing in the models (based on having a bivariate association with the outcome at a p-value of < 0.2). Those with the highest strength of association included gastroenterologist consultation within the prior year (odds ratio [OR] 0.217, 95% confidence interval [CI] 0.114–0.415), CD-related hospitalization within prior year (OR 3.06, 95% CI 1.73–5.41), intra-abdominal surgery during admission (OR 0.206, 95% CI 0.0490–0.865) previous exposure to steroids (OR 1.86, 95% CI (1.05–3.30)), history of bowel resection (OR 1.72, 95% CI (0.989-3.00)), current use of steroids (OR 1.63, 95% CI (0.904–2.95)), and new CD diagnosis (OR 0.377, 95% CI (0.114–1.24)).

**Fig. 1:**
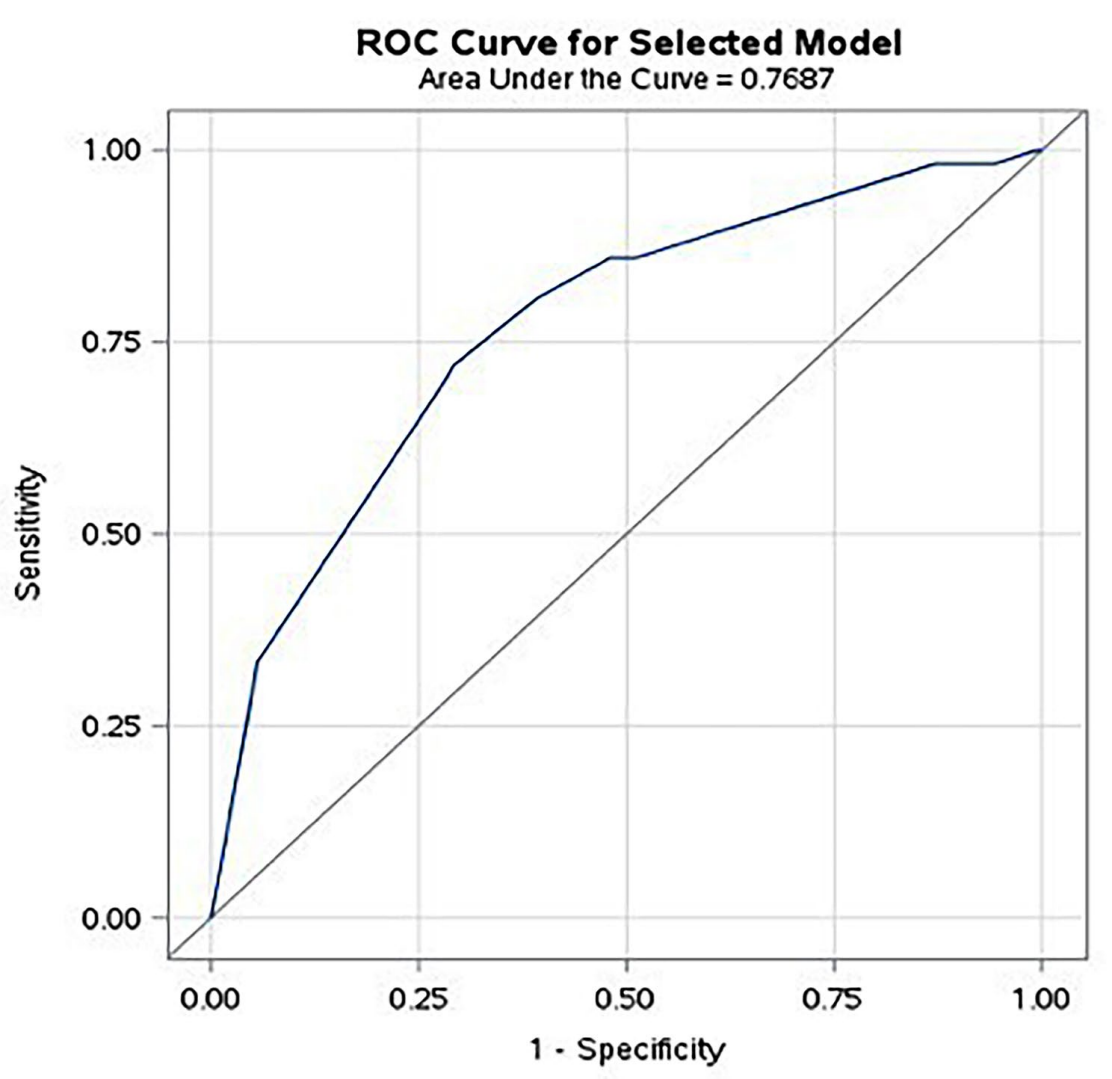
Receiver operating curve for risk of 90-day re-hospitalization among hospitalized persons with CD. Notes: ROC = receiver operator curve

Variables that were retained in the final multivariable logistic regression model, along with their adjusted OR (aOR) and 95% CI, are shown in Table 2. These included gastroenterologist consultation within the prior year, CD-related hospitalization within prior year, intra-abdominal surgery during admission, and new CD diagnosis. The goodness-of-fit p-value was 0.990 (non-significant difference between observed and expected values). The model c-statistic value was 0.769 and the optimism-corrected c-statistic value (based on 200 bootstrapped samples) was 0.726. The model receiver operator curve (ROC) is shown in Fig. 1. Model performance summary statistics are shown in Table 3. Based on the optimal probability cut point of 0.127 (corresponding to a maximal J-index of 0.428), the sensitivity, specificity, PPV, NPV, PLR, NLR, FPR and FNR of the model for predicting likelihood of 90-day hospital re-admission were 71.9% (CI 58.5–83.0), 70.9% (66.5–75.0), 23.2% (CI 17.2–30.1), 95.4% (CI 92.6–97.3), 2.47 (CI 1.99–3.96), 0.396 (CI 0.260–0.600), 29.1% and 28.1%, respectively.

**Table 2:** Multivariate logistic regression analysis for risk of 90-day re-hospitalization among hospitalized persons with CD

Variable	Adjusted odds ratio (95% Confidence Interval)
Gastroenterologist Visit Within Prior Year	0.185 (0.095–0.360)
CD-related hospitalization Within Prior Year	3.27 (1.76–6.08)
Intra-abdominal Surgery During Index Hospitalization	0.216 (0.0500–0.934)
New diagnosis of CD at Index Hospitalization	0.327 (0.0950–1.13)
Optimism-corrected C-statistic Value 0.726 Goodness-of-fit Test P-value 0.990	

Notes: The above variables are the four candidate predictors with the highest strength of association in the final multivariate logistic regression model for the outcome of 90-day re-hospitalization

**Table 3:** Diagnostic test characteristics of multivariable model for 90-day re-hospitalization among hospitalized persons with CD

Variable	Statistic
Optimal Probability Cut Point (J-index)	0.127 (0.428)
Sensitivity	71.9
Specificity	70.9
Positive Predictive Value	23.2
Negative Predictive Value	95.4
% Test positive	33.8
% Test negative	66.2
False Positivity Rate	29.1
False Negativity Rate	28.1

## Discussion

In this study of individuals with CD admitted to a tertiary care hospital for an acute CD-related indication, we observed an 11% rate of acute hospital re-admission within 90 days of discharge. Our final multivariable model demonstrated good discrimination, calibration, and diagnostic performance. Hospitalization within the prior year, gastroenterologist consultation within the prior year, intra-abdominal surgery during index hospitalization, and new diagnosis of CD during index hospitalization were independently associated with 90-day rehospitalization, all of which should be easily attainable at the point of care to apply the model in clinical practice. Application of our model would assign roughly one-third of individuals into a high-risk category, of which more than 70% would be destined for re-admission within 90 days. While the PPV of our model was low at 23%, less than 30% of predicted re-hospitalizations were misclassified (FPR 29%, FNR 28%), demonstrating strong potential for cost-effectiveness of the model in clinical practice if targeted outpatient interventions were adopted for those predicted to be at high-risk of hospital readmission.

CD-related hospitalization within one year prior to the index admission was strongly predictive of subsequent early re-admission, while gastroenterologist care within the preceding year was strongly protective against early re-admission in our model, demonstrating the predictive value of historical health services utilization and specialist care. Both of these factors have been associated with risks of adverse IBD-related outcomes in previous studies [6–10, 23−25]. One study based on U.S. Veteran’s Affairs data reported that a lack of scheduled follow-up with a gastroenterologist after hospital discharge increases risk of re-admission [23]. This could be explained by a lesser propensity for outpatient intervention that could mitigate severe disease recurrence or complication. Surgical intervention during index hospitalization was also protective against re-hospitalization, which supports previous studies [6, 8, 10]. This highlights the importance of appropriate surgical management in select patients who are at high risk of complications or who are failing medical therapy.

To a lesser degree, new diagnosis of CD at the time of index admission was also predictive of a lower risk of 90-day hospital re-admission. This may relate to greater potential for treatment response and fewer disease-related complications early in the disease course of CD. Early aggressive medical treatment has been shown improve CD prognosis as compared to delayed intervention [18, 26–28]. Additionally, there may be greater propensity towards close monitoring of newly diagnosed patients following hospital discharge. Interestingly, age at CD diagnosis, disease phenotype, and prior or current treatments did not significantly influence re-admission risk in the presence of the aforementioned model variables, even though prior studies have identified these as predictive variables [13–17]. In our study, anatomical CD distribution, prior CD surgery, current or previous steroids exposure, and discharge on biologic therapy were weakly associated with 90-day readmission risk but were no longer sufficiently predictive in the presence of the retained model variables.

This is the first study to develop a clinical prediction model to identify individuals with CD who are at increased risk of 90-day hospital re-admission. We elected to study 90-day re-hospitalization, as we hypothesize that 30-day re-hospitalization may be more reflective of premature discharge, and is likely too small of a timeframe to allow for interventions that reduce re-admission. Other groups have studied risk factors for hospital re-admission among individuals with IBD, such as younger age, penetrating disease, chronic pain, opioid and steroid use; however, individual risk factors considered in isolation are less likely to adequately risk stratify individuals as compared to a model that considers the collective contribution of multiple risk factors and protective factors [6–10]. While some groups have modeled 30-day re-hospitalization risk, this may be more reflective of inadequate treatment during hospital admission as opposed to gradual recurrence of symptoms, and leaves little window of opportunity to intervene to prevent re-admission [7, 29].

Our study has several limitations. This study was conducted in a cohort of individuals hospitalized in a single tertiary care hospital system, which may limit its generalizability, particularly to rural and remote jurisdictions. Our model has also yet to be externally validated, so we are unaware of its performance in independent cohorts. The retrospective nature of data collection may have resulted in inaccurate and incomplete data for some variables, which may have impacted model validity and performance. We were also unable to capture some variables that have been previously shown to be associated with hospital re-admission for CD, such as smoking, chronic pain, and mental health [7, 30]. Additionally, we were unable to evaluate factors in the immediate post-discharge setting that may have influenced outcomes, such as medication compliance and outpatient healthcare contacts. Finally, we do not know for certain how much better our model would fare as compared to the current standard of clinician judgement for predicting re-admission risk. Notably, beyond an individual’s gastroenterologist accommodating a patient for follow-up shortly following discharge, there are no systematic approaches in place at most institutions to adequately identify this population for targeted outpatient intervention.

Despite its limitations, as our model displays good performance metrics with relatively low misclassification, and as the model variables are easily attainable at the point of care, we are confident that our model could be useful to clinical practice. Application of our model would likely provide a more cost-effective and reliable approach to triaging individuals for close outpatient monitoring and intervention post-discharge than the current approach, which is based entirely on clinician judgement. However, as our model may still misclassify close to 30% of individuals who are destined for re-admission, vigilance is still required to recognize those with a complex disease or other risk factors for a disabling disease course who may merit closer follow-up. Importantly, our model requires validation in local jurisdictions prior to application in practice, as health care utilization may differ considerably across jurisdictions. In particular, it is unknown whether our findings would be relevant in lesser serviced regions, particularly rural or remote communities, where other health services factors, including hospital practice and timely access to outpatient specialist care, may dictate the reliance on hospital-based resources [31]. Future studies should also focus on implementing such models into clinical practice and determining if they can truly reduce risk of re-hospitalization and associated healthcare costs. This may include strategies such as utilizing multidisciplinary teams and nurse practitioners to ensure adequate and timely follow-up as well as ensuring timely access to surgical interventions for advanced CD to prevent further disease complications. More research into modifiable risk factors for re-hospitalization is needed.

## Conclusions

Clinical and health services variables at the time of discharge have the potential to improve identification of persons with CD at risk of early re-hospitalization, thereby permitting targeted outpatient post-discharge intervention. Application of the model to our reference cohort would earmark one-third of persons for early post-discharge intervention, with the potential to benefit more than 70% of persons destined for early re-hospitalization. Although the PPV of our model was low, it incorrectly predicted early re-hospitalization in less than 30% of patients. Future efforts will focus on externally validating this model in other jurisdictions across Ontario and Canada to test its validity and generalizability and testing model utility in clinical practice to reduce re-hospitalizations among CD patients.

### Electronic supplementary material

Below is the link to the electronic supplementary material.

Supplementary Material 1

## Data Availability

The data underlying this article are available in the article and in its online supplementary material.
