# Supplementary material for: Clinical and health care utilization variables can predict 90-day hospital re-admission in adults with Crohn’s disease for point of care risk evaluation
Source: BMC Gastroenterol. 2024 May 17;24:172. doi: 10.1186/s12876-024-03226-7 (PMC11102236; doi:10.1186/s12876-024-03226-7)
Supplement: Supplementary file 1 — Supplementary Material 1 [file 12876_2024_3226_MOESM1_ESM.docx]

**Supplementary File**

**CLINICAL AND HEALTH CARE UTILIZATION VARIABLES CAN PREDICT 90-DAY HOSPITAL RE-ADMISSION IN ADULTS WITH CROHN’S DISEASE FOR POINT OF CARE RISK EVALUATION**

Dziegielewski C*^1^, Gupta S*^2^, Begum J^3,4^, Pugliese M^3,4^, Lombardi J^5^, Kelly E^3,6^, McCurdy JD^1,3,6^, Sy R^1,3,6^, Saloojee N^1,6^, Ramsay T^3^, Benchimol EI^7,8,9,10^, Murthy SK^1,3,4,6^

*co-first authors

^1^Department of Medicine, University of Ottawa, Ottawa, ON, Canada;

^2^Department of Medicine, University of Toronto, Toronto, Ontario, ON, Canada;

^3^Ottawa Hospital Research Institute, University of Ottawa, Ottawa, ON, Canada;

^4^ICES uOttawa, Ottawa, Ontario, Canada

^5^Department of Medicine, McMaster University, Hamilton, Ontario, ON, Canada;

^6^The Ottawa Hospital IBD Centre, Ottawa, ON, Canada

^7^SickKids Inflammatory Bowel Disease Centre, Division of Gastroenterology, Hepatology and Nutrition, The Hospital for Sick Children, Toronto, Ontario, Canada.

^8^Child Health Evaluative Sciences, SickKids Research Institute, Toronto, Ontario, Canada.

^9^ICES, Toronto, Ontario, Canada.

^10^Department of Paediatrics and Institute of Health Policy, Management and Evaluation, University of Toronto, Toronto, Ontario, Canada.

Corresponding author: Sanjay Murthy, The Ottawa Hospital IBD Centre, Department of Medicine, University of Ottawa, 501 Smyth Rd, Ottawa, ON, Canada, K1H 8L6. Email: [smurthy@toh.ca](mailto:smurthy@toh.ca).

**Supplemental Table 1:** Distribution of Candidate Predictors Among Persons Hospitalized and Re-hospitalized for a CD-related indication at The Ottawa Hospital

| **Variable** | **Total cohort (n=524)** | **Not Re-hospitalized (n=467)** | **Re-hospitalized (n=57)** | **OR (95% CI)** | **P value** | |  |
| --- | --- | --- | --- | --- | --- | --- | --- |
| ***Pre-Admission Characteristics*** | | | | | |  | |
| Sex, n (%)  Female  Male | 292 (55.7%)  232 (44.3%) | 264 (56.4%)  203 (43.5%) | 28 (49.1%)  29 (50.9%) | Reference  0.742 (0.428 – 1.29) | 0.289 | |  |
| Age at CD Diagnosis (years; mean±SD) | 30.6 ±16.0 | 30.6±16.3 | 30.8±14.1 | 1.00 (0.984 – 1.02) | 0.921 | |  |
| Charlson Index, n (%)  0-1  ≥ 2 | 465 (88.7%)  59 (11.3%) | 417 (89.3%)  50 (10.7%) | 48 (84.2%)  9 (15.8%) | Reference  1.56 (0.724 – 3.38) | 0.255 | |  |
| Rurality, n (%)  No  Yes | 467 (89.1%)  57 (10.9%) | 418 (89.5%)  49 (10.5%) | 49 (86.0%)  8 (14.0%) | Reference  1.39 (0.624 – 3.11) | 0.419 | |  |
| Disease Duration (years; mean±SD) | 9.96±10.8 | 9.80±10.7 | 11.2±11.3 | 1.01 (0.988 – 1.04) | 0.346 | |  |
| Disease Distribution, n (%)  Missing  Ileal  Ileocolonic  Colonic | 21 (4.0%)  197 (37.6%)  171 (32.6%)  135 (25.8%) | 15 (3.2%)  177 (37.9%)  148 (31.7%)  127 (27.2%) | 6 (10.5%)  20 (35.1%)  23 (40.4%)  8 (14.0%) | Reference  1.38 (0.727 – 2.60)  0.557 (0.238 – 1.31) | 0.327  0.178 | |  |
| History of Fibrostenotic or Penetrating Disease, n (%)  Missing  No  Yes | 5 (1.0%)  303 (57.8%)  216 (41.2%) | 4 (0.9%)  274 (58.7%)  189 (40.5%) | 1 (1.8%)  29 (50.9%)  27 (47.4%) | Reference  1.35 (0.774 – 2.35) | 0.290 | |  |
| History of Perianal Fistulizing Disease or Perianal Surgery, n (%)  No  Yes | 398 (76.0%)  126 (24.0%) | 353 (75.6%)  114 (24.4%) | 45 (78.9%)  12 (21.1%) | Reference  0.826 (0.422 – 1.62) | 0.576 | |  |
| History of Bowel Resection, n (%)  Missing  No  Yes | 4 (0.8%)  334 (63.7%)  186 (35.5%) | 4 (0.9%)  304 (65.1%)  159 (34.0%) | 0 (0.0%)  30 (52.6%)  27 (47.4%) | Reference  1.72 (0.989 – 3.00) | 0.0549 | |  |
| History of Extra-intestinal Manifestations, n (%)  Missing  No  Yes | 6 (1.1%)  411 (78.4%)  107 (20.4%) | 5 (1.1%)  366 (78.4%)  96 (20.6%) | 1 (1.8%)  45 (78.9%)  11 (19.3%) | Reference  0.932 (0.464 – 1.87) | 0.843 | |  |
| Past Exposure to Steroids, n (%)  Missing  No  Yes | 6 (1.1%)  251 (47.9%)  267 (51.0%) | 6 (1.3%)  231 (49.5%)  230 (49.3%) | 0 (0.0%)  20 (35.1%)  37 (64.9%) | Reference  1.86 (1.05 – 3.30) | 0.0343 | |  |
| Past Exposure to Immunomodulator, n (%)  Missing  No  Yes | 5 (1.0%)  230 (43.9%)  289 (55.2%) | 5 (1.1%)  203 (43.5%)  259 (55.5%) | 0 (0.0%)  27 (47.4%)  30 (52.6%) | Reference  0.871 (0.502 – 1.51) | 0.623 | |  |
| Past Exposure to Biologic, n (%)  Missing  No  Yes | 5 (1.0%)  306 (58.4%)  213 (40.6%) | 5 (1.1%)  274 (58.7%)  188 (40.3%) | 0 (0.0%)  32 (56.1%)  25 (43.9%) | Reference  1.14 (0.654 – 1.98) | 0.647 | |  |
| CD Hospitalization Within Prior 3 Months, n (%)  No  Yes | 489 (93.3%)  35 (6.7%) | -- | -- | Reference  1.40 (0.521 – 3.77) | 0.504 | |  |
| CD Hospitalization Within Prior Year, n (%)  No  Yes | 404 (77.1%)  120 (22.9%) | 372 (79.7%)  95 (20.3%) | 32 (56.1%)  25 (43.9%) | Reference  3.06 (1.73 – 5.41) | 0.0001 | |  |
| Gastroenterologist Visit Within Prior Year, n (%)  No  Yes | 242 (46.2%)  282 (53.8%) | 198 (42.4%)  269 (57.6%) | 44 (77.2%)  13 (22.8%) | Reference  0.217 (0.114 – 0.415) | <.0001 | |  |
| ***Hospital Admission Characteristics*** | | | | | |  | |
| Age During Index Hospitalization (years; mean±SD) | 40.7±16.5 | 40.5±16.6 | 42.1±15.8 | 1.01 (0.990 – 1.02) | 0.482 | |  |
| New CD Diagnosis During Index Hospitalization, n (%)  No  Yes | 461 (88.0%)  63 (12.0%) | -- | -- | Reference  0.377 (0.114 – 1.24) | 0.109 | |  |
| Admitting Service, n (%)  Gastroenterology  Other Medicine Service  Surgery | 189 (36.1%)  133 (25.4%)  202 (38.5%) | 173 (37.0%)  117 (25.0%)  177 (37.9%) | 16 (28.1%)  16 (28.1%)  25 (43.9%) | Reference  1.42 (0.664 – 3.02)  1.53 (0.788 – 2.96) | 0.369  0.210 | |  |
| Intra-abdominal Catastrophe During Index Hospitalization, n (%)  No  Yes | 483 (92.2%)  41 (7.8%) | -- | -- | Reference  0.627 (0.187 – 2.10) | 0.450 | |  |
| Clostridium Difficile Colitis, n (%)  No  Yes | 512 (97.7%)  12 (2.3%) | -- | -- | Reference  2.83 (0.743 – 10.8) | 0.127 | |  |
| WBC (mean±SD) | 11.6±5.37 | 11.5±5.39 | 11.8±5.18 | 1.01 (0.962 – 1.06) | 0.682 | |  |
| Hemoglobin (mean±SD) | 123.1±24.9 | 123.3±24.3 | 121.3±29.3 | 1.00 (0.986 – 1.01) | 0.574 | |  |
| TPN, n (%)  No  Yes | 496 (94.7%)  28 (5.3%) | -- | -- | Reference  0.982 (0.287 – 3.36) | 0.977 | |  |
| Surgical Consult, n (%)  No  Yes | 243 (46.4%)  281 (53.6%) | 217 (46.5%)  250 (53.5%) | 26 (45.6%)  31 (54.4%) | Reference  1.04 (0.596 – 1.80) | 0.903 | |  |
| Length of Hospital Stay (days; mean±SD) | 8.94±11.6 | 9.13±11.8 | 7.39±8.86 | 0.983 (0.952 – 1.02) | 0.287 | |  |
| ICU Admission During Index Hospitalization, n (%)  No  Yes | 511 (97.5%)  13 (2.5%) | -- | -- | Reference  0.678 (0.0870 – 5.31) | 0.711 | |  |
| Extra-intestinal Manifestations During Index Hospitalization, n (%)  No  Yes | 477 (91.0%)  47 (9.0%) | -- | -- | Reference  0.973 (0.369 – 2.57) | 0.956 | |  |
| Steroid Use During Index Hospitalization, n (%)  Missing  No  Yes | 2 (0.4%)  394 (75.2%)  128 (24.4%) | 2 (0.4%)  356 (76.2%)  109 (23.3%) | 0 (0.0%)  38 (66.7%)  19 (33.3%) | Reference  1.63 (0.904 – 2.95) | 0.104 | |  |
| Immunomodulator Use During Index Hospitalization, n (%)  Missing  No  Yes | 2 (0.4%)  398 (76.0%)  124 (23.7%) | 2 (0.4%)  358 (76.7%)  107 (22.9%) | 0 (0.0%)  40 (70.2%)  17 (29.8%) | Reference  1.42 (0.775 – 2.61) | 0.255 | |  |
| Biologic Use During Index Hospitalization, n (%)  Missing  No  Yes | 1 (0.2%)  389 (74.2%)  134 (25.6%) | 1 (0.2%)  345 (73.9%)  121 (25.9%) | 0 (0.0%)  44 (77.2%)  13 (22.8%) | 0.842 (0.439 – 1.62) | 0.607 | |  |
| Narcotic Use During Index Hospitalization, n (%)  Missing  No  Yes | 2 (0.4%)  447 (85.3%)  75 (14.3%) | 2 (0.4%)  401 (85.9%)  64 (13.7%) | 0 (0.0%)  46 (80.7%)  11 (19.3%) | Reference  1.50 (0.738 – 3.04) | 0.264 | |  |
| Fibrostenotic or Penetrating Disease During Index Hospitalization, n (%)  Missing  No  Yes | 1 (0.2%)  310 (59.2%)  213 (40.6%) | 0 (0.0%)  278 (59.5%)  189 (40.%) | 1 (1.8%)  32 (56.1%)  24 (42.1%) | Reference  1.10 (0.630 – 1.93) | 0.731 | |  |
| Perianal Disease or Perianal Surgery During Index Hospitalization, n (%)  No  Yes | 446 (85.1%)  78 (14.9%) | 398 (85.2%)  69 (14.8%) | 48 (84.2%)  9 (15.8%) | Reference  1.08 (0.508 – 2.30) | 0.839 | |  |
| Intestinal Disease Flare During Index Hospitalization, n (%)  No  Yes | 162 (30.9%)  362 (69.1%) | 141 (30.2%)  326 (69.8%) | 21 (36.8%)  36 (63.2%) | Reference  0.741 (0.418 – 1.32) | 0.306 | |  |
| Proximal Small Bowel or Gastroduodenal involvement During Index Hospitalization, n (%)  Missing  No  Yes | 7 (1.3%)  472 (90.1%)  45 (8.6%) | 3 (0.6%)  425 (91.0%)  39 (8.4%) | 4 (7.0%)  47 (82.5%)  6 (10.5%) | Reference  1.39 (0.560 – 3.46) | 0.477 | |  |
| Intra-abdominal Surgery During Index Hospitalization, n (%)  No  Yes | 452 (86.3%)  72 (13.7%) | -- | -- | Reference  0.206 (0.0490 – 0.865) | 0.0309 | |  |
| ***Hospital Discharge Characteristics*** | | | | | |  | |
| Discharge Service, n (%)  Gastroenterology  Other Medicine Service  Surgery | 188 (35.9%)  129 (24.7%)  207 (39.5%) | 171 (36.6%)  113 (24.2%)  183 (39.2%) | 17 (29.8%)  16 (28.1%)  24 (42.1%) | Reference  1.41 (0.666 – 2.98)  1.32 (0.685 – 2.54) | 0.371  0.408 | |  |
| Discharge with Steroids, n (%)  Missing  No  Yes | 4 (0.8%)  167 (31.9%)  353 (67.4%) | 3 (0.6%)  147 (31.5%)  317 (67.9%) | 1 (1.8%)  20 (35.1%)  36 (63.2%) | Reference  0.835 (0.467 – 1.49) | 0.542 | |  |
| Discharge with Ileostomy or Colostomy, n (%)  Missing  No  Yes | 1 (0.2%)  492 (93.9%)  31 (5.9%) | 1 (0.2%)  435 (93.1%)  31 (6.6%) | 0 (0.0%)  57 (100.0%)  0 (0.0%) | Reference  <.001 (<.001 - >999.999) | 0.972 | |  |
| Discharge with Biologic, n (%)  Missing  No  Yes | 4 (0.8%)  350 (66.8%)  170 (32.4%) | 4 (0.9%)  307 (65.7%)  156 (33.4%) | 0 (0.0%)  43 (75.4%)  14 (24.6%) | Reference  0.641 (0.340 – 1.21) | 0.168 | |  |
| Discharge with Narcotics, n (%)  Missing  No  Yes | 6 (1.1%)  375 (71.6%)  143 (27.3%) | 6 (1.3%)  338 (72.4%)  123 (26.3%) | 0 (0.0%)  37 (64.9%)  20 (35.1%) | Reference  1.49 (0.830 – 2.66) | 0.183 | |  |

Notes: Intra-abdominal catastrophe is defined as either abdominal perforation, toxic megacolon, fulminant colitis, or intra-abdominal sepsis. ICU=intensive care unit; WBC=white blood cell counts; TPN=total parenteral nutrition.

-- Not reported due to small numbers of events in one or more categories
